# Development and Validation of an Esophageal Squamous Cell Carcinoma Risk Prediction Model for Rural Chinese: Multicenter Cohort Study

**DOI:** 10.3389/fonc.2021.729471

**Published:** 2021-08-30

**Authors:** Junming Han, Lijie Wang, Huan Zhang, Siqi Ma, Yan Li, Zhongli Wang, Gaopei Zhu, Deli Zhao, Jialin Wang, Fuzhong Xue

**Affiliations:** ^1^Department of Biostatistics, School of Public Health, Cheeloo College of Medicine, Shandong University, Jinan, China; ^2^Institute for Medical Dataology, School of Public Health, Cheeloo College of Medicine, Shandong University, Jinan, China; ^3^School of Public Health, Shandong First Medical University & Shandong Academy of Medical Sciences, Jinan, China; ^4^Cancer Prevention and Treatment Center, Feicheng People’s Hospital, Feicheng, China; ^5^Centre for Health Management and Policy Research, School of Public Health, Cheeloo College of Medicine, Shandong University, Jinan, China; ^6^Department of Health Statistics, School of Public Health, Weifang Medical University, Weifang, China; ^7^Department of Human Resource, Shandong Cancer Hospital and Institute, Shandong First Medical University and Shandong Academy of Medical Sciences, Jinan, China

**Keywords:** esophageal squamous cell carcinoma, risk prediction, individualized assessment, China, Cox proportional hazards model

## Abstract

**Background:**

There are rare prediction models for esophageal squamous cell carcinoma (ESCC) for rural Chinese population. We aimed to develop and validate a prediction model for ESCC based on a cohort study for the population.

**Methods:**

Data of 115,686 participants were collected from esophageal cancer (EC) early diagnosis and treatment of cancer program as derivation cohort while data of 54,750 participants were collected as validation cohort. Risk factors considered included age, sex, smoking status, alcohol drinking status, body mass index (BMI), tea drinking status, marital status, annual household income, source of drinking water, education level, and diet habit. Cox proportional hazards model was used to develop ESCC prediction model at 5 years. Calibration ability, discrimination ability, and decision curve analysis were analyzed in both derivation and validation cohort. A score model was developed based on prediction model.

**Results:**

One hundred eighty-six cases were diagnosed during 556,949.40 person-years follow-up in the derivation cohort while 120 cases from 277,302.70 in the validation cohort. Prediction model included the following variables: age, sex, alcohol drinking status, BMI, tea drinking status, and fresh fruit. The model had good discrimination and calibration performance: *R* ^2^, D statistic, and Harrell’s C statistic of prediction model were 43.56%, 1.70, and 0.798 in derivation cohort and 45.19%, 1.62, and 0.787 in validation cohort. The calibration analysis showed good coherence between predicted probabilities and observed probabilities while decision curve analysis showed clinical usefulness. The score model was as follows: age (3 for 45–49 years old; 4 for 50–54 years old; 7 for 55–59 years old; 9 for 60–64 years; 10 for 65–69 years), sex (5 for men), BMI (1 for ≤25), alcohol drinking status (2 for alcohol drinkers), tea drinking status (2 for tea drinkers), and fresh fruit (2 for never) and showed good discrimination ability with area under the curve and its 95% confidence interval of 0.792 (0.761,0.822) in the deviation cohort and 0.773 (0.736,0.811) in the validation cohort. The calibration analysis showed great coherence between predicted probabilities and observed probabilities.

**Conclusions:**

We developed and validated an ESCC prediction model using cohort study with good discrimination and calibration capability which can be used for EC screening for rural Chinese population.

## Introduction

Cancer is a particularly important health problem among noncommunicable diseases and is also the first or second cause of premature mortality in more than 90 countries across the world (1). Esophageal cancer (EC) remains a major cause of cancer mortality and burden across the world despite reductions in age-standardized incidence and mortality rates and is of great difference between incidence rate of EC among different races and regions (2, 3). As the most populous country across the world, more than 23% of new cancer cases, about 30% of cancer deaths, and about half of the new cases of EC worldwide occur in China (4). Also, ESCC accounts for 90% of EC cases and is the main type of EC in China (5–7).

Endoscopy examination is one of the main inspection modality for ESCC or precursor lesion and endoscopic screening programs have been performed for high-risk populations across the world (8, 9). Several screening programs for EC have been carried out in China, as follows: a community-based endoscopic screening for esophageal cancer for residents aged 25–65 years from six villages in Hua County, Henan Province, China which was carried out from 2007 (10); regional organized esophageal cancer screening programs since 2005 in endemic areas in China for 40–69-year-old adults (11, 12); endoscopic screening for esophageal cancer in China (ESECC), a cluster RCT, carried out from January 2012 to September 2016 for people aged 45–69 years (13, 14); “Taihang Anti-cancer Campaign” including three stations in Linxian in Henan Province, Cixian in Hebei Province and Yangchen in Shanxi Province for people aged ≥40 years old (15); a population-based case-control study from October 2010 to September 2013 in Taixing for participants aged 40–84 years (16); and the Esophageal, Stomach, Liver Cancer Screening Program (ESLCSP) carried out in four provinces: Jiangsu, Anhui, Shandong, and Henan in China for people aged 40 to 69 years (17). For people with special situations, such as, a history of head-and-neck cancers (18, 19), high-risk population-tylosis, disease onset for Achalasia, injury history of caustic esophageal injury (11), and Barrett’s esophagus may be advised to have regular endoscopic examinations (8, 20).

In 2006, a national early diagnosis and treatment guideline for EC was introduced and stated that cancer screening was to be performed by endoscopy accompanied with iodine staining and indicative biopsy in high-risk population in China (21, 22). Since then, rural residents aged 40 to 69 years participated in the cancer screening at the national esophageal cancer early diagnosis and treatment base. The prevalence of ESCC and precancerous lesions, risk factors associated with ESCC and precancerous lesions, the survival of patients with precancerous lesion, early cancer, and advanced cancer after treatment had been reported basically based on the cancer screening data (23–27).

To the best of our knowledge, there are few risk prediction models for ESCC. Previous prediction models were developed in China, Japan, Sweden, and Iran population based on case-control studies (28–31) and were also developed based on the cohort study in China and from the Nord-Trondelag Health Study (17, 32). These models show good discriminative performance as area under the receiver operating curve (AUC) ranged from 0.76 to 0.81 and were mainly based on risk factors such as age, sex, smoking, alcohol drinking, body mass index (BMI), diet habits like consumption of fresh fruits, salted food, alarming symptoms of retrosternal pain, alarming symptoms of back pain, alarming symptoms of neck pain, family history of upper gastrointestinal cancer, and disease history of esophagitis or peptic ulcer. The previous models have the following limitations: (1) the factors used in previous models are difficult to obtain or quantified such as alarming symptoms; (2) lack of external validation; and (3) most of them were based on case-control studies.

The aim of this paper was to develop a risk prediction model of ESCC for rural Chinese population and tested its performance in both derivation cohort and validation cohort.

## Materials and Methods

### Study Population

This study was based on EC cancer screening data based on early diagnosis and treatment program from centers of the cancer prevention and treatment in Shandong province. **Supplementary Figure S1** shows the screening procedure, and it had been described in detail elsewhere (22). Briefly, before cancer screening, an informed consent form would be signed and a questionnaire including personal basic information, risk factors of esophageal cancer, family history, and simple physical examination should be filled. The participants were then assessed whether they were suitable for endoscopy and endoscopy examination was performed.

The derivation cohort came from cancer prevention and treatment centers except in Feicheng city in Shandong province, and the data were collected from 2012 to 2018. The validation cohort came from the centers in Feicheng city, and data were collected from 2012 to 2019.

Inclusion criteria of the study were as follows: (a) participants enrolled in the early diagnosis and treatment of cancer programs from 2012 in Shandong province; (b) participants aged older than 39 and younger than 70 years; (c) participants did not have a history of cancer recorded by the cancer registry and were not diagnosed of cancer at baseline; (d) participants were not diagnosed with dysplasia or worse at baseline by pathologic diagnosis; (e) participants who had filled the baseline questionnaire survey correctly; and (f) participants who had signed informed consent.

### Data Collection and Candidate Predictors of ESCC

The data that included questionnaire information, endoscopic examination results, and pathologic diagnosis of participants were collected in the derivation cohort and validation cohort. The questionnaire information included age, sex, height, weight, marital status, education level, source of drinking water, smoking, drinking, diet habits, history of gastrointestinal diseases and age diagnosed, family history of cancer, etc. The results of endoscopic examination included location, size and shape of lesions, and endoscopic diagnosis. Biopsies were taken at the site of the lesion, and the pathological diagnosis was made by biopsy. Pathological diagnosis was recorded as each participant’s highest-level diagnosis. Endoscopy, specimen treatment, and pathologic diagnosis are referred to Chinese cancer screening, early diagnosis, and early treatment technology published by the People’s Health Publishing House.

The risk factors to be studied were selected according to previous research results, review articles, etc. (17, 28, 31, 33–42). The candidate variables were age, sex, BMI, smoking status, alcohol drinking status, tea drinking status, annual household income, marital status, education level, source of drinking water and diet habit of fresh fruit, high-temperature food, fried food, and pickled food. Age was divided into six categories: 40–44, 45–49, 50–54, 55–59, 60–64, or 65–69 years old. BMI was divided into two categories: 0 (>25 kg/m^2^) and 1 (≤25 kg/m^2^). Smoking status was divided into two categories: 0 (nonsmokers) and 1 (smokers). Alcohol drinking status was also divided into two categories: 0 (nonalcohol drinkers) and 1 (alcohol drinkers). Tea drinking status was also divided into two categories: 0 (nontea drinkers) and 1 (tea drinkers). Annual household income was divided into four categories (low, medium-low, medium-high, and high) according to the quantile. Marital status was divided into two categories: 0 (single, divorced, or widowed participants) and 1 (married participants). Education level was divided into two categories: 0 (junior high school or lower level) and 1 (senior high school or higher level). Source of drinking water was divided into two categories: 0 (untreated water: water obtained from spring, well, or river) and 1 (treated water: running water or purified water). Diet habit of fresh fruit and high-temperature food was divided into two categories: 0 (yes, had eaten the food) and 1 (never). Diet habit of pickled food and fried food was divided into two categories: 0 (less than twice a week on average) and 1 (two times or more per week on average).

### Outcome Definitions

New ESCC were diagnosed according to the International Classification of Diseases, 10th version as well as histology (ICD-O morphology codes). The follow-up time of the derivation cohort and validation cohort was up to December 31, 2020. The outcome was confirmed by cancer registration data.

### Statistical Analysis

#### Model Derivation

The risk predictors in the prediction model were determined using three steps. The first step was fitting full model with all candidate variables using a multivariable Cox regression model. The variable was eliminated from the full model if its coefficient was greater than 0.90 and less than 1.10 and was not statistically significant at level 0.01 in the second step (43, 44). In the third step, the candidate variables eliminated in the second step was added into the multivariable model one by one and if performance statistics of the model were improved significantly, they would be added to the model again. We examined interactions between variables and age in the final model and included significant interactions at level 0.05 into the final model. We computed a 5-year risk prediction model of ESCC in the derivation cohort and used complete case for all analyses.

#### Assessment of Model Performance

*R*^2^ value, D statistic and its 95% confidence interval (95% CI), Harrell’s C statistic and its 95% CI, calibration curve and decision curve analysis were used to test the model performance (43, 44). *R* ^2^ value means the variation the model explained, and higher value of *R* ^2^ is better. D statistic and Harrell’s C statistic are measures of discriminative ability, and higher values mean better discrimination performance. Harrell’s C is similar to AUC but considers the censored nature of the survival data. We also calculated AUC and its 95% CI as well as Somers’ D statistic for comparison with other models previously established. Calibration curves were used to assessed the agreement of predicted probability and observed probability (45). Calibration-in-the-large (A) and calibration slope (B) was calculated (46). The closer A is to 0 and the closer B is to 1, the better the model calibration performance is. Decision curve analysis was performed for clinical use and higher net-benefit indicated better clinical usefulness.

#### External Validation

We tested the performance of the 5-year ESCC prediction model in validation cohort. *R* ^2^ value, D statistic and 95% CI, Harrell’s C statistic and 95% CI, calibration curve, and decision curve were used to do the external validation.

#### Score Model

The score model was derived based on the prediction model. The risk scores of each risk predictor were calculated by its corresponding coefficient dividing the minimum coefficient from the prediction model. The risk scores were finally derived by rounding to the nearest 1 (47). The total scores of participants were obtained by calculating the scores of each risk factor. AUC (95% CI), Somers’ D statistics, and calibration curve were used to test the performance of this score model both in the derivation cohort and validation cohort.

We comprehensively estimated the performance of each cutoff value in the score model. We calculate proportion of high-risk population whose total score was more than the cutoff value. The validity was evaluated by sensitivity, specificity, Youden’s index, and likelihood ratio including positive likelihood ratio and negative likelihood ratio. We also calculate accuracy rate and predictive value including positive predictive value and negative predictive value. The number needed to be screened and the predicted risk of developing ESCC within 5 years were also calculated. The candidate cutoff value should have a higher Youden’s index and a similar proportion of high-risk population to that reported before.

Participants diagnosed with dysplasia were reentered into the derivation cohort and validation cohort to perform sensitivity analyses. The *R* ^2^ value, D statistic and 95% CI, and Harrell’s C statistic and 95% CI were also used for sensitivity analyses. We also did the predictor selection progress using forward stepwise method, and the AIC optimization criterion was used.

The analyses were performed using R software version 4.0.4 (https://www.r-project.org/). Package *survival* was used for Cox regression analysis. Package *cmprsk* and package *ggDCA* were used for decision curve analysis.

## Results

### Study Population

**Figure 1:**
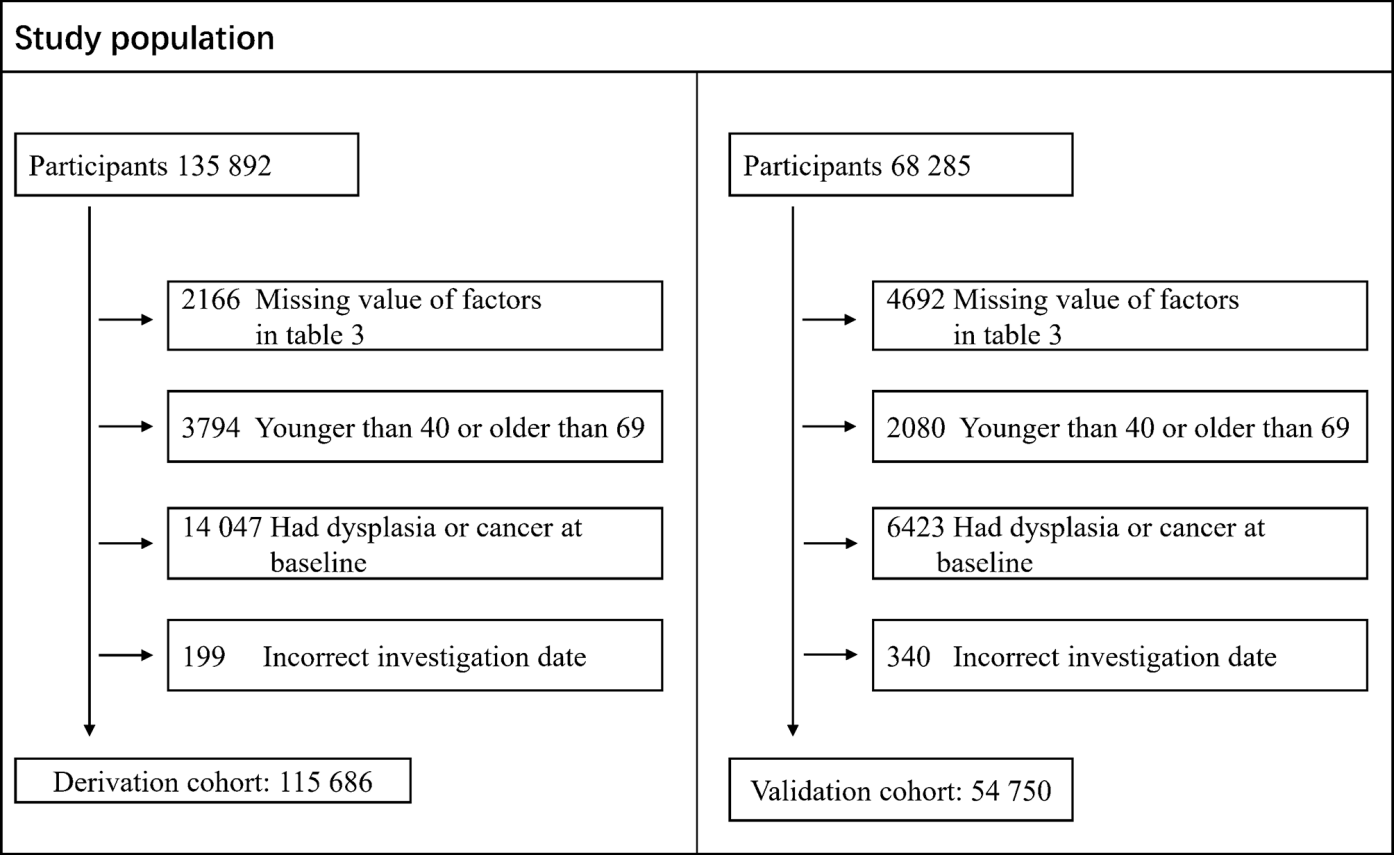
Flow chart of the study population data collation including derivation cohort and validation cohort.

One hundred fifteen thousand six hundred eighty-six individuals were included in the derivation cohort after excluding 14,047 participants with any cancer diagnosis or dysplasia, 3,794 participants aged older than 69 or younger than 40 years, 2,166 participants with missing value of risk factors in **Table 3**, and 199 participants with incorrect investigation data as indicated in **Figure 1**. **Supplementary Figures S2–S6** show the cumulative incidence of ESCC by age, sex, BMI, alcohol drinking status, and tea drinking status in the derivation cohort. For the validation cohort, 54,750 individuals were included after excluding 6,423 participants with any cancer diagnosis or dysplasia, 2,080 participants aged older than 69 or younger than 40 years, 4,692 participants with missing value of risk factors in **Table 3**, and 340 participants with incorrect investigation data as indicated in **Figure 1**.

### Baseline Characteristics

**Table 1** shows the characteristics in the derivation cohort and validation cohort. Women accounted for 57.70% of the derivation cohort and 60.42% of the validation cohort, participants younger than 50 years old accounted for 27.32% and 31.90%, respectively, BMI greater than 25 accounted for 35.21% and 45.68%, respectively, smokers accounted for 21.37% and 39.19%, respectively, alcohol drinkers accounted for 21.34% and 41.33%, respectively, tea drinkers accounted for 22.24% and 71.95%, respectively, low level of annual household income accounted for 17.13% and 33.51%, respectively, participants married accounted for 95.85% and 95.61%, respectively, participants drinking treated water accounted for 70.63% and 28.88%, respectively, participants with senior high school or higher education level accounted for 49.61% and 60.66%, respectively, participants never has fresh fruit accounted for 13.29% and 7.49%, respectively, participants had high-temperature food accounted for 82.90% and 43.92%, respectively, participants with low level of fried food accounted for 34.53% and 91.41%, respectively, and participants with high level of pickled food intake accounted for 53.81% and 28.64%, respectively.

**Table 1:** Baseline characteristics of participants in the derivation cohort and validation cohort.

Characteristics	Derivation Cohort *n* = 115, 686	Validation Cohort *n* = 54, 750
**Sex**		
Women	66,753 (57.70)	33,079 (60.42)
Men	48,933 (42.30)	21,671 (39.58)
**Age (years)**		
40–44	10,851 (9.38)	6,610 (12.07)
45–49	20,749 (17.94)	10,854 (19.83)
50–54	25,771 (22.28)	12,083 (22.07)
55–59	21,069 (18.20)	10,352 (18.90)
60–64	22,449 (19.41)	9,186 (16.78)
65–69	14,797 (12.79)	5,665 (10.35)
**BMI**		
**>25**	40,728 (35.21)	25,010 (45.68)
**≤25**	74,958 (64.79)	29,740 (54.32)
**Smoke status**		
No	90,963 (78.63)	33,294 (60.81)
Yes	24,723 (21.37)	21,456 (39.19)
**Alcohol drink status**		
No	91,000 (78.66)	32,123 (58.67)
Yes	24,686 (21.34)	22,627 (41.33)
**Tea drink status**		
No	89,958 (77.76)	15,359 (28.05)
Yes	25,728 (22.24)	39,391 (71.95)
**Annual household income**		
High	25,705 (22.22)	11,032 (20.15)
Medium-high	40,384 (34.91)	16,301 (29.77)
Medium-low	16,773 (14.50)	9,069 (16.56)
Low	19,820 (17.13)	18,348 (33.51)
Missing	13,004 (11.24)	0
**Marital status**		
Single, divorced, or widowed	3,460 (2.99)	2,339 (4.27)
Married	110,884 (95.85)	52,347 (95.61)
Missing	1,342 (1.16)	64 (0.12)
**Source of drinking water**		
Treated water	81,711 (70.63)	15,813 (28.88)
Untreated water	29,858 (25.81)	34,861 (63.67)
Missing	4,117 (3.56)	4,076 (7.45)
**Education level**		
Junior high school or lower level	56,728 (49.04)	20,370 (37.21)
Senior high school or higher level	57,390 (49.61)	33,211 (60.66)
Missing	1,568 (1.36)	1,169 (2.13)
**Fruit**		
Yes	98,433 (85.09)	50,647 (92.51)
Never	15,370 (13.29)	4,103 (7.49)
Missing	1,883 (1.62)	0
**High-temperature food**		
Never	16,006 (13.84)	30,705 (56.08)
Yes	95,900 (82.90)	24,045 (43.92)
Missing	3,780 (3.26)	0
**Fried food**		
Low level	39,945 (34.53)	50,048 (91.41)
High level	71,492 (61.80)	4,702 (8.59)
Missing	4,249 (3.67)	
**Pickled food**		
Low level	49,498 (42.79)	39,069 (71.36)
High level	62,246 (53.81)	15,681 (28.64)
Missing	3,942 (3.40)	0

Values are numbers (percentages).

BMI, body mass index.

### Incidence Rate

**Table 2** shows the incidence rate in the derivation cohort and validation cohort. A total of 186 new cases of ESCC were diagnosed during the 556,949.40 person-years follow-up in the derivation cohort. The incidence rate of ESCC was 33.40 per 100,000 person-years. The incidence rate of ESCC was 13.52 per 100,000 person-years for women and 61.35 for men. The average follow-up time was 4.81 years. The incidence rate of ESCC increased with age.

**Table 2:** Incidence rates of ESCC per 100,000 person-years in the derivation cohort and validation cohort.

Group	Derivation Cohort			Validation Cohort		
	Incident Cases	Person-years	Rate	Incident Cases	Person-years	Rate
**Total**	186	556,949.40	33.40	120	277,302.70	43.27
**Sex**						
Women	44	325,479.40	13.52	33	168,592.10	19.57
Men	142	231,470.00	61.35	87	108,710.60	80.03
**Age (years)**						
40–44	3	55,280.32	5.43	1	37,085.83	2.70
45–49	12	101,865.19	11.78	7	56,561.54	12.38
50–54	21	122,157.31	17.19	21	59,417.26	35.34
55–59	33	102,536.12	32.18	28	52,063.92	53.78
60–64	57	106,951.42	53.30	40	45,338.65	88.22
65–69	60	68,159.00	88.03	23	26,835.48	85.71

In the validation cohort, a total of 120 new cases of ESCC were diagnosed during the 277,302.70 person-years follow-up. The incidence rate of ESCC was 43.27 per 100,000 person-years in the validation cohort. The incidence rate of ESCC was 19.57 per 100,000 person-years for women and 80.03 for man. The average follow-up time was 5.06 years. The incidence rate of ESCC also increased with age.

### ESCC Prediction Model

**Table 3** shows the adjusted hazard ratios of risk factors in multivariable regression model. The final prediction model included risk factors, such as, age, sex, BMI, alcohol drinking status, tea drinking status, and fresh fruit after selecting procedure. There was no statistically significant interaction between age and other variables in the prediction model. In the prediction model, men were associated with 236% increased risk of ESCC, BMI lower than 25 was associated with 30% increased risk of ESCC, alcohol drinkers were associated with 59% increased risk of ESCC, tea drinkers were associated with 51% increased risk of ESCC, and never had fresh fruit were associated with 52% increased risk of ESCC in derivation cohort.

**Table 3:** Adjusted hazard ratio of risk factors associated with ESCC in the multivariable Cox model.

Predictor Variables	Crude HR (95% CI)	Adjust HR (95% CI)
**Age (years)**		
40–44	Reference	Reference
45–49	2.17 (0.61,7.67)	2.20 (0.62,7.80)
50–54	3.15 (0.94,10.57)	3.00 (0.89,10.10)
55–59	5.89 (1.81,19.22)	5.49 (1.68,17.97)
60–64	9.76 (3.06,31.16)	9.27 (2.90,29.62)
65–69	16.06 (5.03,51.20)	15.04 (4.71,47.97)
**Sex**		
Women	Reference	Reference
Men	4.52 (3.22,6.34)	3.36 (2.33,4.86)
**BMI**		
>25	Reference	Reference
≤25	1.44 (1.04,1.99)	1.30 (0.93,1.80)
**Drink status**		
No	Reference	Reference
Yes	3.17 (2.37,4.24)	1.59 (1.14,2.20)
**Tea drink status**		
Never	Reference	Reference
Yes	2.06 (1.52,2.79)	1.51 (1.09,2.09)
**Fresh fruits**		
Yes	Reference	Reference
Never	1.38 (0.95,2.02)	1.52 (1.04,2.23)

BMI, body mass index; HR, hazard ratio.

### Validation of Prediction Model

#### Discrimination Performance

**Table 4** shows the discrimination performance of the prediction model in the derivation cohort and validation cohort. The prediction model explained 43.56% of the variation, the D statistic was 1.70 and Harrell’s C was 0.798 in the derivation cohort, and the corresponding values were 45.19%, 1.62 and 0.787 in the validation cohort.

**Table 4:** Statistics of the performance of developed prediction model of ESCC.

Statistic	Derivation Cohort	Validation Cohort
**D statistic**	1.70 (1.47,1.94)	1.62 (1.32,1.92)
**Harrell’s C**	0.798 (0.769,0.827)	0.787 (0.751,0.823)
***R*^2^ (%)**	43.56	45.19

Prediction model: age, sex, BMI, alcohol drinking status, and tea drinking status. D statistic and Harrell’s C: evaluate model discrimination ability, higher values mean better discrimination ability; R^2^: the variance of the model interpreted, higher value means better.

#### Calibration Performance

**Figure 2:**
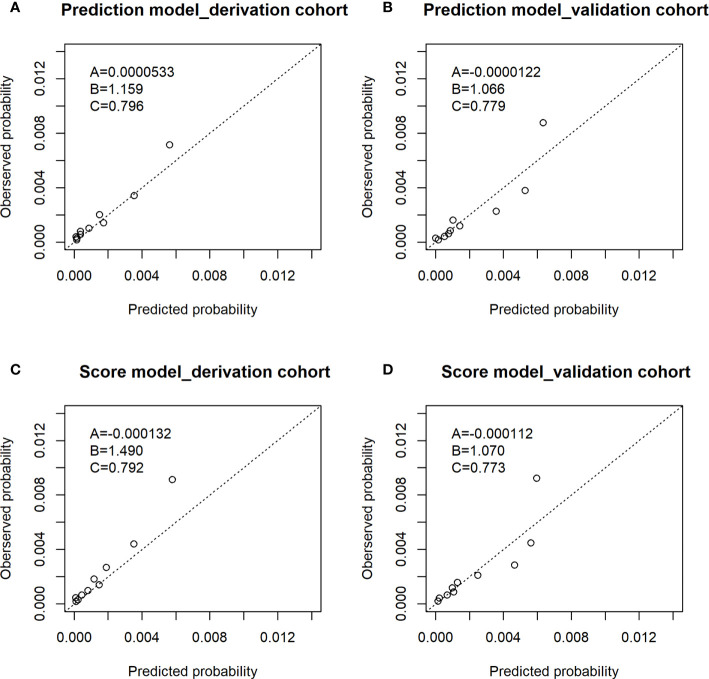
Observed proportion of ESCC and predicted risk of ESCC within 5 years in the derivation cohort and validation cohort. **(A)** The calibration plot of prediction model of derivation cohort; **(B)** the calibration plot of prediction model of validation cohort; **(C)** the calibration plot of score model of derivation cohort; **(D)** the calibration plot of score model of validation cohort. The A,B,C in the figures: (A) The intercept. (B) The slope. (C) The AUC value.

**Figure 2** shows observed proportion of ESCC and predicted risk of ESCC within 5 years for the prediction model in both the derivation cohort and validation cohort. The horizontal axis in the figure was the predicted probability of ESCC for 10 groups divided according to the quantile of predicted probability. The ordinate axis represented the observed proportion of ESCC corresponding to 10 groups. There was agreement between predicted risk and observed risk in the derivation cohort as the points in **Figure 2** was close to the line with slope of 1 and intercept of 0 and A was 0.0000533 and B was 1.159. There was an agreement between the predicted risk and observed risk in the validation cohort as the points in **Figure 2** was close to the line with slope of 1 and intercept of 0 and A was −0.0000122 and B was 1.066.

### Decision Curve

**Figure 3:**
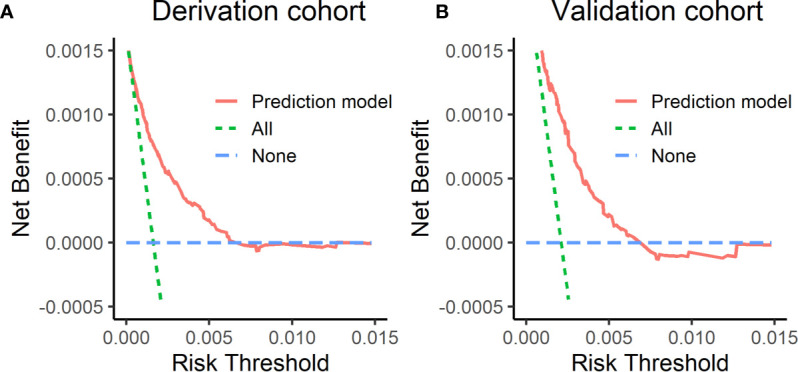
Decision curves in the derivation cohort and validation cohort. **(A)** The decision curve in the derivation cohort, **(B)** the decision curve in the validation cohort. The horizontal axis of this picture was threshold probability. The ordinate axis was the net benefit after the advantages were subtracted by the disadvantages.

**Figure 3** shows decision curves in the derivation cohort and validation cohort. The horizontal axis of this picture was threshold probability. When one’s risk of ESCC reached a certain threshold, it was defined as positive and some intervention measures were taken. The ordinate axis was the net benefit (NB) after the advantages were subtracted by the disadvantages. Prediction model had a higher NB than treating all the participants as high- or low-risk population. It indicated that the model developed in this study was clinical usefulness.

### Score Model

#### Score Model

**Table 5** shows the risk scores of each risk factor in the score model: age (3 for 45–49 years old; 4 for 50–54 years old; 7 for 55–59 years old; 9 for 60–64 years; 10 for 65–69 years), sex (5 for men), BMI (1 for ≤25), alcohol drinking status (2 for alcohol drinkers), tea drinking status (2 for tea drinkers), and fresh fruit (2 for never). **Supplementary Table S1** shows the score model has good discrimination ability with AUC (95%CI) of 0.792 (0.761, 0.822) in the deviation cohort and 0.773 (0.736, 0.811) in the validation cohort. There was an agreement between predicted risk and observed risk in the derivation cohort as the points in **Figure 2** was close to the line with slope of 1 and intercept of 0 and A was −0.000132 and B was 1.490. There was an agreement between the predicted risk and observed risk in the validation cohort as the points in **Figure 2** was close to the line with slope of 1 and intercept of 0 and A was −0.000112 and B was 1.070.

**Table 5:** Coefficient of predictor variables from prediction model and its corresponding score.

Predictor Variables	Coefficient	Score
**Age**		
**40–44**	Reference	0
**45–49**	0.79	3
**50–54**	1.10	4
**55–59**	1.70	7
**60–64**	2.23	9
**65–69**	2.71	10
**Sex**		
**Women**	Reference	0
**Men**	1.21	5
**BMI**		
>25	Reference	0
≤25	0.26	1
**Drink status**		
**No**	Reference	0
**Yes**	0.46	2
**Tea drink status**		
**No**	Reference	0
**Yes**	0.41	2
**Fresh fruit**		
**Yes**	Reference	0
**Never**	0.42	2

BMI, body mass index.

#### Effectiveness of the Score-Based Prediction Model of ESCC

**Supplementary Table S2** shows the performances of each cutoff value. As the cutoff value increased, the predicted risk of ESCC, specificity, and accuracy rate increased while the proportion of high-risk individuals and the sensitivity in detecting ESCC decreased. The Youden’s index first increased and then decreased as the cutoff value increased and reached its maximum when the cutoff value was 13. The total risk scores ranged from 0 to 22. Considering high-risk individuals, sensitivity, and Youden’s index, the candidate cutoff values of 12 and 13 could be selected as the criteria for identifying high-risk individuals for cancer screening.

The score of participants was used as a predictor to predict the incidence of ESCC. The regression coefficient was 0.245 as the score was a continuous variable. We then divided the participants into five categories using quantiles. Compared with the lowest quintile, the HR (95% CI) of the highest quintile was 46.47 (11.48, 188.07), the HR (95% CI) of the fourth quintile was 16.87 (4.07, 70.01), the HR (95% CI) of the third quintile was 7.30 (1.73, 30.89) and the HR (95% CI) of the second quintile was 1.92 (0.52, 10.47).

**Supplementary Table S3** shows the result of sensitivity analysis. Sensitivity analysis showed that the model was stable without deletion of dysplasia at baseline with *R* ^2^ of 43.82%, D statistic of 1.69, and Harrell’s C of 0.799 (0.769, 0.828) in the derivation cohort and *R* ^2^ of 35.38%, D statistic of 1.33, and Harrell’s C of 0.729 (0.700, 0.758) in the validation cohort. **Supplementary Table S4** shows the forward stepwise selection and the best combination of risk factors was age, sex, BMI, alcohol drinking status, tea drinking status, and fresh fruit as shown in **Supplementary Table S4**.

## Discussion

We developed and validated an individual 5-year risk prediction model of ESCC using cohort study for population aged 40 to 69 years and provided an efficient tool for rural Chinese in this study. The prediction model included six variables, such as, age, sex, BMI, alcohol drinking status, tea drinking status, and diet habit of fresh fruit and showed good discrimination and calibration ability in both the derivation cohort and validation cohort. Decision curve analysis showed that the prediction model had clinical usefulness. A score model was derived from the prediction model and also showed good discrimination and calibration ability in both the internal validation and external validation.

Since 2006, as the first batch of pilot, Feicheng city, a high incidence area of ESCC which the validation cohort came from, began the EC cancer screen program, and evidence had shown that the screen is associated with a reduction of the incidence of ESCC (48). It was reported that, from 2006 to 2016, the detection rate of precancerous lesions of ESCC was 6.81% in the Feicheng screening program (26). The research based on the screening data of Feicheng showed that alcohol drinking, men, tea drinking, etc. were the risk factors for the occurrence and development of precancerous lesions (24, 49, 50). After that, EC screening programs were carried out in other centers in rural China which the derivation cohort came from. The baseline characteristics and incidence rate of derivation cohort and validation cohort were different; however, the model established in this study performs well in both cohorts. The model was still stable after sensitivity analysis. Therefore, the model may be suitable for popularization and application. During the clinical practice in the EC screening program, the candidates for the screening were selected mainly based on age and whether they had contraindications of endoscopic screening due to being inconvenient of the evaluation tool. In this study, besides age, the risk factors in this study were divided into two groups, which was easy to obtain, can be accurately measured, and easy to apply in practical work.

There were rare prediction models for ESCC based on the cohort study. Chen et al. used logistic regression model and developed an ESCC prediction model based on multicenter cohort study in rural China without validation (17). The model included risk factors such as pain of back was difficult to obtain and quantify accurately. Wang et al. developed an ESCC prediction model based on Nord-Trondelag Health Study in Norway and validated the model in a cohort based on UK Biobank (32). The prediction model included five risk factors: age, sex, BMI, smoking status, and drinking status. The AUC of the 5-year ESCC prediction model was 0.76 in derivation cohort and was 0.70 in validation cohort. The classification criteria of the following risk factors such as drinking status are different. The etiology of ESCC is multifaceted, and there are great differences in various populations. According to the results of this study, tea drinking was a risk factor in the prediction model and that is meaningful because it is a very traditional habit among Chinese population.

This study focused attention on questionnaire-related risk factors other than genetic or other risk factors. Previous studies had shown that the risk of ESCC in men was several times higher than that in women, and the ratio varied greatly among different populations (51). In this study, incidence rate of ESCC in men was about three times of the incidence rate in women. Alcohol drinking was an important risk factor of ESCC and a dose-response relationship has been found (52–55). People with lower weight have a higher risk of ESCC (32). In this study, tea drinking is an important risk factor for ESCC, and the reason may be the high temperature of the tea (56, 57). Chinese traditional health-keeping philosophy believes that drinking hot liquids in the heat will increase sweating and benefit health. Drinking tea is a very common and popular habit in China and is also a traditional way of leisure and entertainment. The relationship between tea drinking and cancer is still unclear while green tea is generally considered to be cancer resistant because it contains catechin or polyphenols (58, 59). According to our study, preventive measures should be carried out for risk factors such as reducing alcohol drinking, reducing hot tea drinking, and keep proper weight.

This paper also has some limitations. (1) The data were collected from one province of China, which could not represent the 40- to 69-year population of China. On the other hand, Shandong is a big province in China and accounting for nearly one-tenth of the population of China. To some extent, it is also representative. (2) Although our cohort was large, the number of outcomes with esophageal cancer is small, which was likely to cause residual bias. This problem could be solved by longer follow-up. (3) There might have a recall bias for the risk factors. (4) We did not obtain the data of the following risk factors: PAH and trace element. We will add related questions to the questionnaire in the near future.

In summary, this paper provides a prediction model for ESCC with good discrimination and calibration in internal verification and external verification. Our prediction model is discriminative as previous established models with AUC of 0.796 in the derivation cohort and higher discriminative with AUC of 0.779 in the validation cohort as shown in the **Supplementary Table S5**. The prediction model offered a reliable and accurate prediction and can be used for screening program for ESCC. In the future, we will conduct more extensive external validation and adjust the model by adding other characteristics of participants.

## Conclusion

In this study, we developed and validated an ESCC prediction model for rural Chinese population based on a multicenter cohort study. The prediction model included five risk factors: age, sex, BMI, alcohol drinking status, tea drinking status, and diet habit of fresh fruit. The prediction model showed good discrimination and calibration ability in both the derivation cohort and validation cohort. Decision curve analysis showed its clinical usefulness. We transferred the prediction model into a score model for clinical use, and the score model also showed good discrimination and calibration ability in the derivation cohort and validation cohort. We provided a simple and useful prediction tool for ESCC and may be used for ESCC cancer screen.

## Data Availability Statement

Data used in this research and related data could be offered upon reasonable request for the corresponding author.

## Ethics Statement

The studies involving human participants were reviewed and approved by Ethics Committee of Public Health, Shandong University. The patients/participants provided their written informed consent to participate in this study.

## Author Contributions

JH and FX designed the study. JH carried out the analysis and wrote the first version of the manuscript. LW provided help in the process of data analysis. HZ, SM, ZW, GZ, YL, DZ, and JW helped with data collection and collation. All the authors participated in the discussion and agreed to publish the model in its current format. All authors contributed to the article and approved the submitted version.

## Funding

This study is funded by the National Key R&D Program of China (2020YFC2003500) from the Ministry of Science and Technology of the People’s Republic of China.

## Conflict of Interest

The authors all declare that the research was conducted in the absence of any commercial or financial relationships that could be construed as a potential conflict of interest.

## Publisher’s Note

All claims expressed in this article are solely those of the authors and do not necessarily represent those of their affiliated organizations, or those of the publisher, the editors and the reviewers. Any product that may be evaluated in this article, or claim that may be made by its manufacturer, is not guaranteed or endorsed by the publisher.

## Abbreviations

A, calibration in large; AUC, area under the receiver operating curve; B, calibration slope; BMI, body mass index; EC, esophageal cancer; ESCC, esophageal squamous cell carcinoma; ESECC, endoscopic screening for esophageal cancer in China; ESLCSP, the Esophageal, Stomach, Liver Cancer Screening Program; NB, net benefit; 95% CI, 95% confidence interval.
