# Supplementary material for: Development and Validation of an Esophageal Squamous Cell Carcinoma Risk Prediction Model for Rural Chinese: Multicenter Cohort Study
Source: Front Oncol. 2021 Aug 30;11:729471. doi: 10.3389/fonc.2021.729471 (PMC8435773; doi:10.3389/fonc.2021.729471)
Supplement: Supplementary file 1 [file DataSheet_1.docx]

Supplementary Material

# Supplementary Figures and Tables

## Supplementary Figures


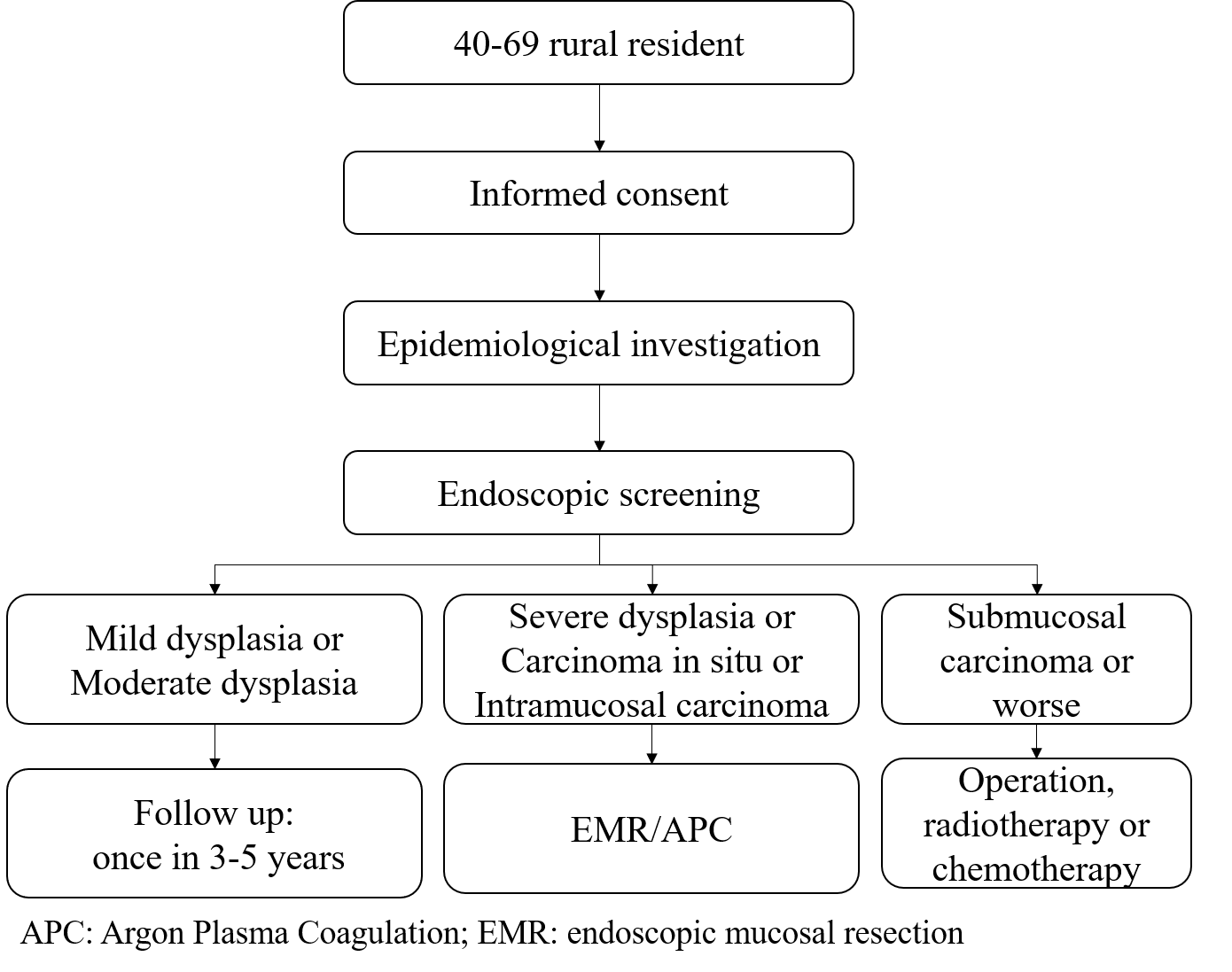


**Supplementary Figure 1.** An overview of the screening procedure of the cancer screening


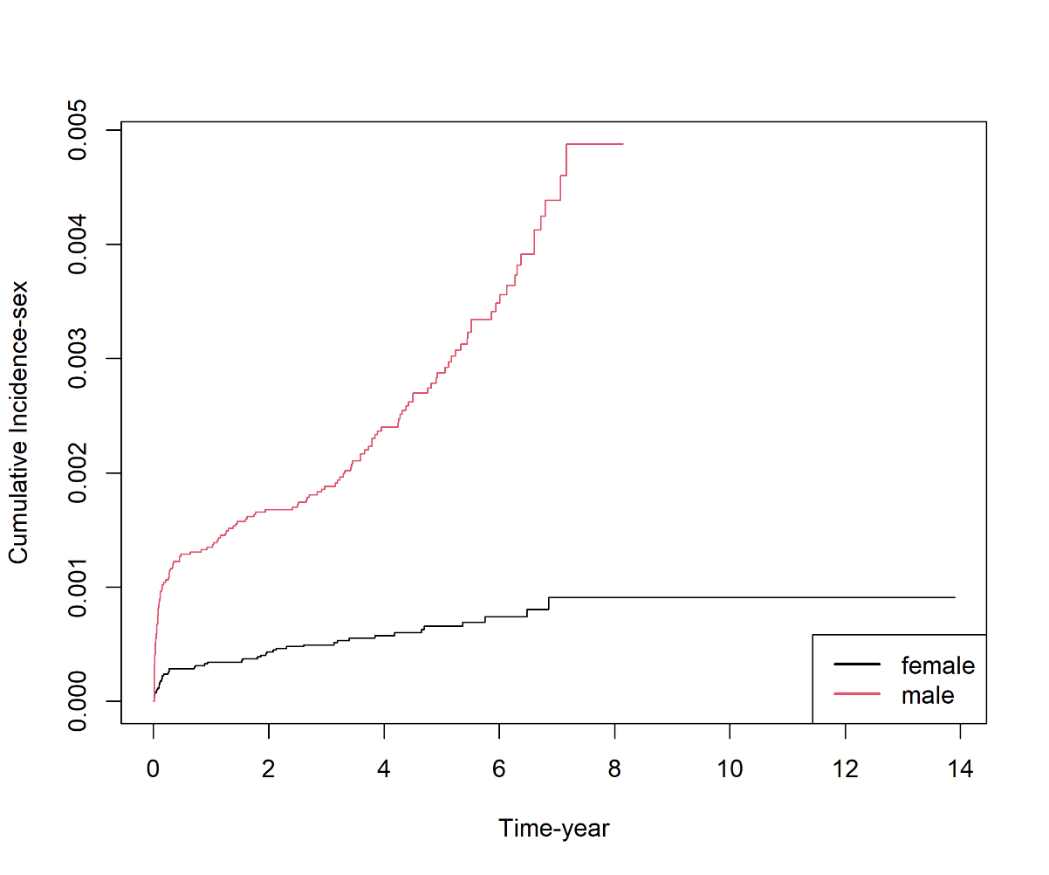


Supplementary Figure 2. Cumulative incidence of ESCC by sex in derivation cohort.


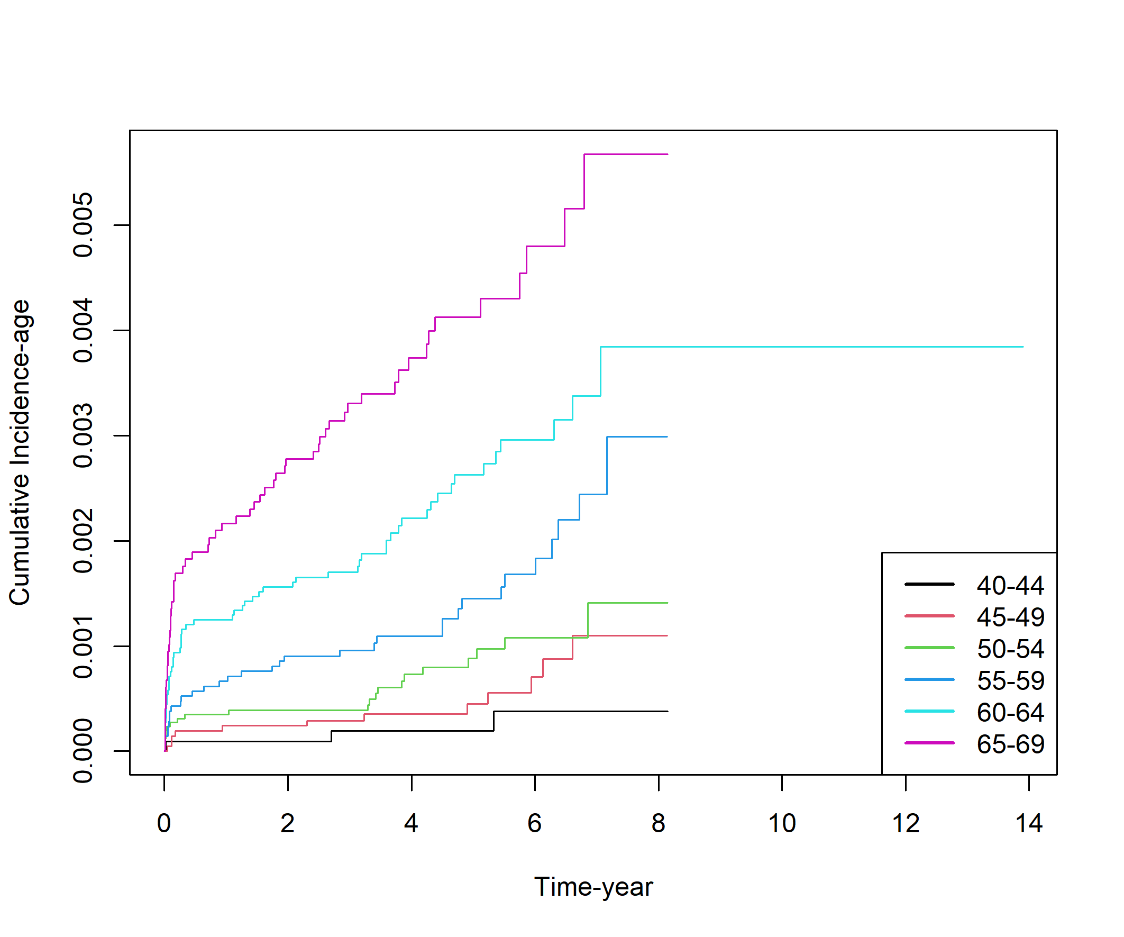


Supplementary Figure 3. Cumulative incidence of ESCC by age in derivation cohort.


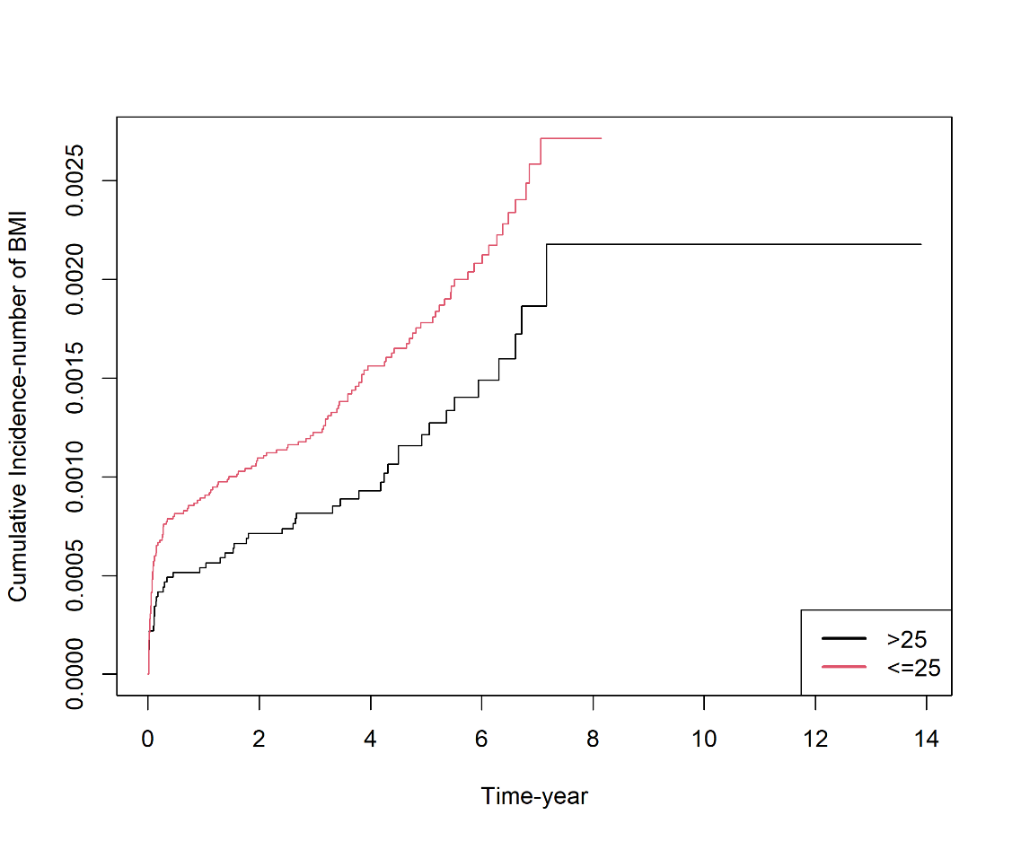


Supplementary Figure 4. Cumulative incidence of ESCC by BMI in derivation cohort.


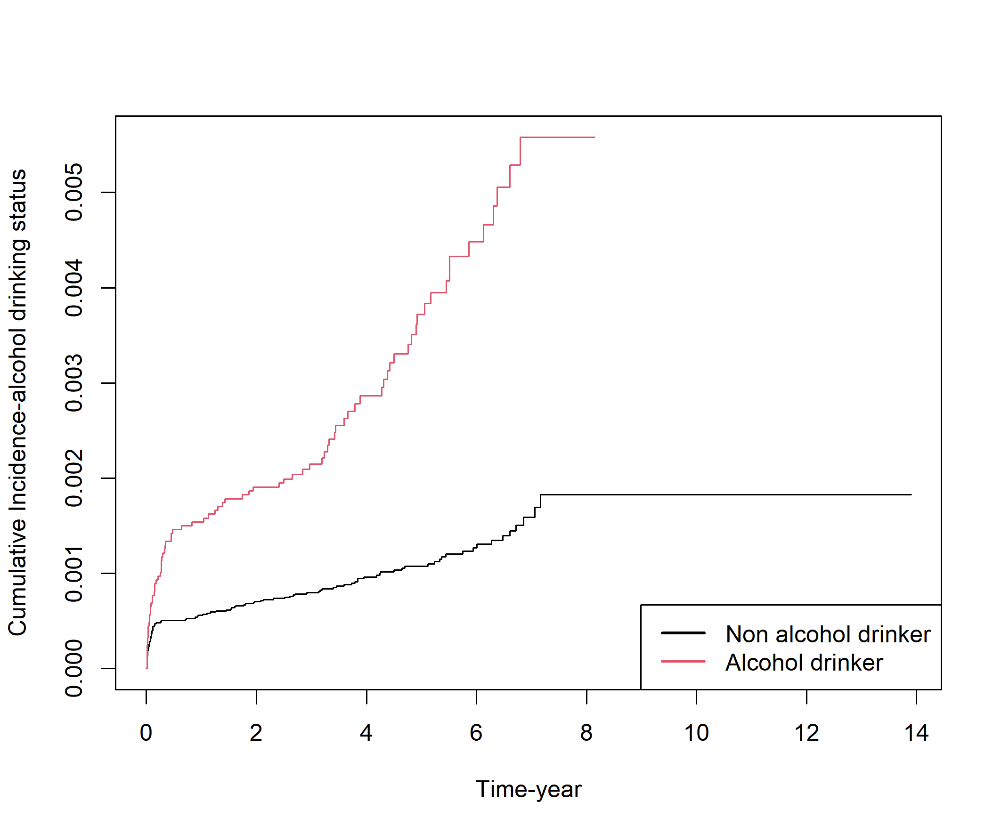


Supplementary Figure 5. Cumulative incidence of ESCC by alcohol drinking status cohort in derivation cohort.


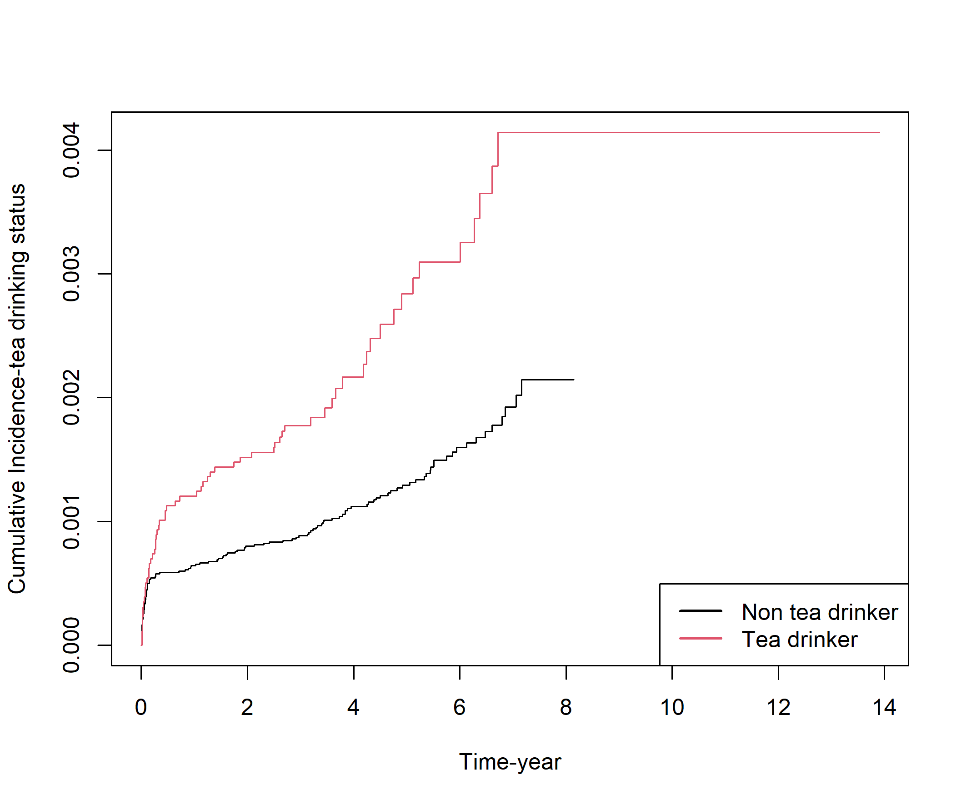


Supplementary Figure 6. Cumulative incidence of ESCC by tea drinking status in derivation cohort.

## Supplementary Tables

**Supplementary Table 1.** Statistics of the performance of the score model of ESCC

|  | **Derivation cohort** | **Ten-fold cross validation** | **Validation cohort** |
| --- | --- | --- | --- |
| **AUC** | 0.792(0.761,0.822) | 0.784(0.753,0.816) | 0.773(0.736,0.811) |
| **Somer's D** | 0.584 | 0.568 | 0.546 |

AUC: aera under the receive operator curve.

**Supplementary Table 2.** The performances of each score as a cut-off value in identifying individuals at a high risk of ESCC.

| **Score** | **Pr** | **Sen** | **Spe** | **Youden's index** | **Accuracy rate** | **PPV** | **NPV** | **+LR** | **-LR2** | **NNS** | **Risk** |
| --- | --- | --- | --- | --- | --- | --- | --- | --- | --- | --- | --- |
| 1 | 0.989 | 1.000 | 0.011 | 0.011 | 0.013 | 0.001 | 1.000 | 1.011 | 0.000 | 754.133 | 0.00010 |
| 2 | 0.963 | 1.000 | 0.037 | 0.037 | 0.039 | 0.001 | 1.000 | 1.039 | 0.000 | 734.207 | 0.00014 |
| 3 | 0.958 | 1.000 | 0.042 | 0.042 | 0.044 | 0.001 | 1.000 | 1.044 | 0.000 | 730.602 | 0.00018 |
| 4 | 0.925 | 1.000 | 0.075 | 0.075 | 0.077 | 0.002 | 1.000 | 1.082 | 0.000 | 705.254 | 0.00023 |
| 5 | 0.842 | 0.988 | 0.159 | 0.146 | 0.160 | 0.002 | 1.000 | 1.174 | 0.079 | 649.997 | 0.00030 |
| 6 | 0.766 | 0.975 | 0.234 | 0.209 | 0.235 | 0.002 | 1.000 | 1.273 | 0.107 | 599.207 | 0.00038 |
| 7 | 0.727 | 0.969 | 0.273 | 0.242 | 0.274 | 0.002 | 1.000 | 1.333 | 0.114 | 572.431 | 0.00050 |
| 8 | 0.673 | 0.969 | 0.328 | 0.296 | 0.329 | 0.002 | 1.000 | 1.441 | 0.095 | 529.641 | 0.00064 |
| 9 | 0.595 | 0.944 | 0.405 | 0.349 | 0.406 | 0.002 | 1.000 | 1.587 | 0.139 | 480.914 | 0.00084 |
| 10 | 0.522 | 0.931 | 0.478 | 0.409 | 0.479 | 0.003 | 1.000 | 1.785 | 0.144 | 427.851 | 0.00108 |
| 11 | 0.400 | 0.831 | 0.600 | 0.431 | 0.600 | 0.003 | 1.000 | 2.079 | 0.281 | 367.475 | 0.00140 |
| 12 | 0.323 | 0.750 | 0.678 | 0.428 | 0.678 | 0.003 | 0.999 | 2.327 | 0.369 | 328.343 | 0.00182 |
| 13 | 0.263 | 0.700 | 0.737 | 0.437 | 0.737 | 0.004 | 0.999 | 2.665 | 0.407 | 286.823 | 0.00236 |
| 14 | 0.218 | 0.644 | 0.783 | 0.427 | 0.783 | 0.004 | 0.999 | 2.964 | 0.455 | 258.060 | 0.00305 |
| 15 | 0.180 | 0.588 | 0.821 | 0.408 | 0.820 | 0.005 | 0.999 | 3.273 | 0.503 | 233.771 | 0.00395 |
| 16 | 0.122 | 0.456 | 0.878 | 0.335 | 0.878 | 0.005 | 0.999 | 3.753 | 0.619 | 203.995 | 0.00512 |
| 17 | 0.085 | 0.350 | 0.915 | 0.265 | 0.915 | 0.006 | 0.999 | 4.137 | 0.710 | 185.190 | 0.00663 |
| 18 | 0.045 | 0.181 | 0.955 | 0.136 | 0.954 | 0.006 | 0.999 | 4.003 | 0.858 | 191.321 | 0.00859 |
| 19 | 0.025 | 0.131 | 0.975 | 0.106 | 0.974 | 0.007 | 0.999 | 5.254 | 0.891 | 146.043 | 0.01112 |
| 20 | 0.011 | 0.056 | 0.989 | 0.045 | 0.987 | 0.007 | 0.999 | 5.022 | 0.954 | 152.749 | 0.01439 |
| 21 | 0.004 | 0.019 | 0.996 | 0.015 | 0.995 | 0.007 | 0.999 | 5.122 | 0.985 | 149.771 | 0.01861 |

Pr: the proportion of high-risk individuals; Sen: sensitivity; Spec: specificity; PPV: positive predictive value; NPV: negative predictive value; +LR: positive likelihood ratio; −LR: negative likelihood ratio; NNS: number needed to screen.

**Supplementary Table 3** Statistics of the sensitivity analysis

| **Statistic** | **Derivation cohort** | **Validation cohort** |
| --- | --- | --- |
| **D statistic** | 1.69 (1.92,1.45) | 1.33(1.13,1.53) |
| **Harrell's C** | 0.799(0.769,0.828) | 0.729 (0.700,0.758) |
| $\boldsymbol{R}^{\boldsymbol{2}}$**(%)** | 43.82 | 35.38 |

D statistic and Harrell's C: evaluate model discrimination ability, higher values mean better discrimination ability; $R^{2}$: the variance the model interpretated, higher value means better.

Supplementary Table 4 Forward stepwise selection

| Stepwise | Df | Deviance | AIC | Harrell's C |
| --- | --- | --- | --- | --- |
|  | NA | NA | 3208.2 |  |
| +Sex | -1 | 69.71 | 3133.9 | 0.675 |
| +Age | -5 | 15.67 | 3074.1 | 0.777 |
| +Tea drinking status | -1 | 4.87 | 3060.5 | 0.789 |
| +Alcohol drinking status | -1 | 4.87 | 3057.6 | 0.794 |
| +Fresh fruit | -1 | 2.31 | 3057.3 | 0.797 |
| +BMI | -1 | 1.97 | 3057.3 | 0.798 |

The AIC optimization criterion is used to select the variables entering the model.

**Supplementary Table 5** Statistics of the prediction model

|  | **Derivation cohort** | **Ten-fold cross validation** | **Validation cohort** |
| --- | --- | --- | --- |
| **AUC** | 0.796(0.766-0.826) | 0.785(0.753-0.816) | 0.779(0.742-0.816) |
| **Somer's D** | 0.592 | 0.569 | 0.558 |

Prediction model: age, sex, BMI, alcohol drinking status, tea drinking status and diet habit of fresh fruit. AUC: aera under the receive operator curve.
